# Neuropsychological and Emotional–Behavioral Profiles in Pediatric Duchenne Muscular Dystrophy: A Single-Center Clinical Study

**DOI:** 10.3390/children13080992

**Published:** 2026-07-27

**Authors:** Rossella D’Alessandro, Francesca Re, Martina Vacchetti, Francesca Sertori, Luca Arletti, Alice Campagna, Giulio Gadaleta, Tiziana Enrica Mongini, Federica Silvia Ricci

**Affiliations:** 1 Section of Child and Adolescent Neuropsychiatry, “Regina Margherita” Children’s Hospital, AO OIRM-S. Anna, Department of Public Health and Pediatric Sciences, University of Turin, 10126 Turin, Italymartina.vacchetti@unito.it (M.V.); francesca.sertori@unito.it (F.S.); luca.arletti@unito.it (L.A.); alice.campagna@edu.unito.it (A.C.); federica.ricci@unito.it (F.S.R.); 2 Neuromuscular Unit, AOU Città Della Salute e Della Scienza di Torino, Department of Neurosciences “Rita Levi Montalcini”, University of Turin, 10126 Turin, Italy; giulio.gadaleta@unito.it (G.G.); tizianaenrica.mongini@unito.it (T.E.M.)

**Keywords:** Duchenne muscular dystrophy, pediatric neuropsychiatric disorders, ADHD, ASD, emotional–behavioral, cognitive functioning

## Abstract

**Highlights:**

**What are the main findings?**

- A multimodal psychometric screening battery was systematically administered in a cross-sectional, single-center study involving children with Duchenne muscular dystrophy (DMD), together with a review of clinical history and cognitive functioning.
- Screening identified attentional difficulties, internalizing vulnerabilities, emotional dysregulation and autism-related features beyond motor impairment. Among children with available cognitive data, nearly half presented a low FSIQ, namely in the Dp140−/Dp71+ subgroup.

**What are the implications of the main findings?**

- These preliminary, exploratory findings support the value of multidomain psychometric screening to identify and diagnose neuropsychiatric and cognitive vulnerabilities in children with DMD.
- The heterogeneous profile observed in the cohort highlights the need for personalized assessments to optimize interventions.

**Abstract:**

**Background**: Duchenne muscular dystrophy (DMD) is an X-linked disorder caused by out-of-frame variants in the *DMD* gene, resulting in dystrophin deficiency and progressive muscle degeneration. Beyond motor involvement, evidence links DMD to cognitive impairment and an emotional–behavioral (EB) burden, potentially related to the altered expression of brain dystrophin isoforms (Dp71, Dp140, Dp427). **Objectives**: To screen for major neurodevelopmental, cognitive and/or EB difficulties in a monocentric cohort of children with DMD. **Methods**: This cross-sectional study included 21 children with DMD. Neuropsychiatric difficulties were assessed using a multimodal psychometric battery. Cognitive, neurodevelopmental and genetic data were retrospectively collected and analyzed. **Results**: In the cohort, attention-deficit/hyperactivity disorder (ADHD)-related findings were predominantly inattentive, with 3/20 children (15.0%) scoring within the clinical range on at least one inattention subscale. For measures assessing autism spectrum disorder (ASD)-related features, scores above the normative cutoff emerged in 7/20 children (35.0%), while only 2/20 (10.0%) scored within the clinical range. Internalizing problems represented the predominant EB difficulties, and emotional dysregulation (ED) emerged as a plausible area of vulnerability in the cohort. Among the 11 of 21 children with available Full-Scale Intelligence Quotient (FSIQ) data, five (45.5%) had an FSIQ below 85. Of these, four of five (80.0%) presented the predicted Dp140−/Dp71+ brain dystrophin isoform expression pattern, whereas one of five (20.0%) presented the Dp140+/Dp71+ pattern. **Conclusions**: Children with DMD showed heterogeneous neuropsychiatric and cognitive features in the employed screening battery. These preliminary findings, if confirmed in larger cohorts, support the potential of a broader neuropsychiatric screening assessment to optimize care pathways.

## 1. Introduction

Duchenne muscular dystrophy (DMD) is the most common and severe form of dystrophinopathy, typically caused by out-of-frame variants in the dystrophin gene (*DMD* gene) [1,2]. Located on the X chromosome, at Xp21.2–p21.1, this is the largest known human gene and encodes the full-length dystrophin protein (Dp427). The disorder is rare, with a pooled global prevalence of approximately 7 cases per 100,000 males and an incidence of between 1:3500 and 1:5000 live male births [2,3,4]. Clinically, DMD has an early onset, typically between 2 and 5 years of age, and follows a progressive course leading to loss of independent ambulation, respiratory failure and cardiomyopathy. Early manifestations include delayed motor milestones, difficulties in running and jumping, frequent falls, a waddling gait, toe walking, lumbar hyperlordosis and Gowers’ sign, often accompanied by speech delays and articulation difficulties, suggesting involvement that extends beyond the muscular system from the earliest stages of development [3,5]. Indeed, beyond its well-established muscular involvement, DMD is increasingly recognized as a multisystem disorder that also affects the central nervous system, particularly during neurodevelopment [6,7]. This dual involvement mirrors the architecture of the dystrophin gene itself, which, through internal promoters, expresses several brain isoforms—including Dp427, Dp140 and Dp71 [7,8,9]—which are implicated in key neurodevelopmental processes, such as synaptic function, neuronal differentiation and myelination. Consequently, a deficiency in these isoforms, especially in the more distal Dp140 and Dp71, has been associated with an increased risk of cognitive and neurobehavioral impairments [6,10]. Approximately one third of children with DMD exhibit cognitive deficits [6], often independently of motor severity, involving language, executive function, working memory, attention and learning abilities [8,11,12]. In parallel, genotype–phenotype correlations indicate that pathogenic variants affecting the more distal brain isoforms confer a higher risk of intellectual disability and psychiatric comorbidities [6,10,13]. This neurobehavioral vulnerability translates into increased rates of autism spectrum disorder (ASD) and attention-deficit/hyperactivity disorder (ADHD), as well as executive dysfunction, affecting inhibitory control, cognitive flexibility and adaptive functioning [8]. Overall, these findings highlight that DMD should be considered a multisystem disorder with significant neurocognitive and neuropsychiatric involvement [6,7,8], for which early identification and targeted interventions are needed to improve children’s outcomes and quality of life [5,14,15,16]. Despite growing awareness of these neuropsychiatric manifestations, few pediatric studies have systematically characterized the full neuropsychiatric profile of DMD using a comprehensive multimodal approach [17,18,19]. Moreover, emotional dysregulation problems, although among the most frequently reported neurodevelopmental features in DMD, remain relatively under-investigated.

To address this gap, a monocentric, cross-sectional observational study was conducted in a pediatric cohort of 21 children with DMD to comprehensively characterize their cognitive profile and to screen for neurodevelopmental and/or emotional–behavioral problems. For this purpose, a psychometric screening battery based on internationally validated age-appropriate tests was developed and administered during routine standard-of-care visits to detect possible neuropsychiatric vulnerabilities, including internalizing problems and ADHD- and ASD-related features. Furthermore, clinical, genetic and neuropsychological data, including available measures of cognitive functioning, were retrospectively extracted from medical records and collected.

## 2. Materials and Methods

A monocentric, cross-sectional observational study was conducted in a cohort of children with genetically confirmed DMD, regularly in follow-up at the Pediatric Neuromuscular Unit of the “Regina Margherita” Children’s Hospital in Turin, Italy, enrolled between September 2024 and August 2025. A minimum age of 5 years was established to ensure the psychometric validity and developmental appropriateness of the neuropsychiatric assessment battery. This study was conducted according to the principles of the Declaration of Helsinki, revised in October 2013. Written informed consent for the Neurodevelopment Internalizing Disorder (NID) study was obtained from the parents of all children included in the study. This study was approved by the Institutional Ethics Committee on 24 September 2021 (approval code: 0097848).

### 2.1. Participants

The study was initially proposed to all 23 eligible children followed at the pediatric Neuromuscular Unit for routine visits during the study period. Of these, 21 children aged 5–17 years agreed to participate and were consecutively recruited. No eligible participant was excluded for reasons other than refusal. For each child, demographic, clinical and genetic data were collected through review of the hospital’s electronic medical records (TrakCare, version 8, InterSystems Corporation, Cambridge, MA, USA, 2011).

### 2.2. Psychometric and Neuropsychological Assessments

To systematically screen for the main neuropsychiatric difficulties in the cohort, a dedicated multidomain psychometric battery was developed, based on internationally validated age-appropriate instruments. Both parent-report questionnaires and clinician-administered semi-structured interviews were proposed during routine standard-of-care visits to maximize screening sensitivity. All instruments used in the study were validated for the age ranges included in the cohort and administered in Italian. Scoring was conducted using Italian normative data for age in all children enrolled.

#### 2.2.1. Parent-Reported Assessments

The following five parent-report questionnaires were completed independently by caregivers and scored against age-adjusted normative data:

1. Child Behavior Checklist (CBCL) [20], available in age-appropriate versions (CBCL 1.5–5 and CBCL 6–18 years). This assesses a broad range of emotional and behavioral problems over the preceding six months through parent report. Items are rated on a 0–2 scale and organized into competence scales, broadband problem scales (internalizing, externalizing and total problems), syndrome scales, Diagnostic and Statistical Manual of Mental Disorders (DSM)-oriented scales and 2007 scales. Scores are expressed as T-scores and classified as normative, borderline or clinical. Additionally, the Dysregulation Profile (DP)—computed as the sum of the T-scores on the attention problems, aggressive behavior, and anxious/depressed subscales—was used as a dimensional measure of emotional dysregulation (ED), with a total T-score ≥ 180 indicating a clinically significant impairment.
2. Conners’ Parent Rating Scale—Revised (CPRS-R) [21]. This consists of 80 items rated on a 0–3 scale and assesses ADHD symptoms and related behavioral domains, including oppositional behavior, cognitive problems/inattention, hyperactivity, anxiety, social difficulties and psychosomatic problems, in children and adolescents aged 3–17 years. Raw scores are converted to age-adjusted T-scores, with scores above 61 considered outside the normal range and significantly problematic scores above 70. The scale also includes two DSM-IV symptom subscales, namely inattention and hyperactivity–impulsivity, specifically designed to assess whether symptom frequency meets the DSM-IV diagnostic criteria for ADHD, with the clinical cutoff set at a score ≥ 6 of 9 on either subscale.
3. ADHD Rating Scale 5th Version (ADHD-RS-V)—parent version [22]. This is an 18-item questionnaire rated on a 0–3 frequency scale, comprising 9 items targeting inattention and 9 targeting hyperactivity–impulsivity. The clinical cutoff is defined as ≥6 items scoring ≥2 on either subscale.
4. Social Communication Questionnaire (SCQ)—Lifetime Form [23]. This is a 40-item yes/no screening instrument for autism spectrum disorder (ASD), evaluating reciprocal social interaction, communication and restricted/repetitive behaviors across the child’s entire developmental history. A total score ≥ 15 indicates a positive screening result and prompts consideration of further diagnostic evaluation.
5. Behavior Rating Inventory of Executive Function—Second Edition (BRIEF-2) [24,25]. This is a 63-item questionnaire assessing executive function behaviors in home and school settings in children aged 5–18 years. Items are scored on a three-point frequency scale (never/sometimes/often) and organized into nine clinical scales that generate three composite indices—the Behavioral Regulation Index (BRI), the Emotional Regulation Index (ERI) and the Cognitive Regulation Index (CRI)—and an overall Global Executive Composite (GEC). T-scores ≥ 70 are considered clinically elevated. The ERI, comprising the shift and emotional control subscales, was specifically used as an additional indicator of ED, complementing the CBCL Dysregulation Profile.

#### 2.2.2. Clinician-Administered Semi-Structured Interviews

To increase the screening sensitivity for the presence of neurodevelopmental disorder-related features in the cohort, standardized semi-structured interviews were additionally administered to one or both caregivers:

1. The Checklist for Autism Spectrum Disorder (CASD) [26], in which the clinician recorded the lifetime presence of each of 30 symptoms corresponding to the DSM-5 ASD criteria, organized across six clinical domains. A total score ≥ 15 is consistent with an ASD diagnosis; scores of 11–14 indicate a borderline or PDD-NOS (pervasive developmental disorder—not otherwise specified) presentation, while scores of 8–10 indicate an at-risk condition.
2. The ADHD screening module of the Kiddie Schedule for Affective Disorders and Schizophrenia, Present and Lifetime Version (K-SADS-PL DSM-5) [27], was administered to assess current ADHD symptomatology according to the DSM-5 criteria. The module explores 18 behaviors (9 inattention, 9 hyperactivity–impulsivity criteria) rated on a 0–3 clinician-assigned scale. A high probability of an ADHD diagnosis is defined by ≥6 threshold-level symptoms (score = 3) on either subscale.

### 2.3. Cognitive Profile, Developmental History and Other Clinical Variables

Clinical and neuropsychological data were additionally retrospectively collected through a review of medical records. Cognitive functioning assessments were performed by district services responsible for the child’s care; consequently, the data included in this study were derived from the assessment reports provided, when available. Not all local services were able to provide the corresponding assessment reports. Cognitive assessments were performed close in time to the study evaluation. Specifically, the most recent cognitive assessment available for each participant was used, in accordance with standard clinical practice, which generally recommends an interval of at least 18–24 months between two formal cognitive assessments to avoid repeated testing over a short period and to reduce practice effects. Evaluations had been administered using age-appropriate standardized instruments: the Wechsler Intelligence Scale for Children (WISC-IV) [28] and the Wechsler Preschool and Primary Scale of Intelligence (WPPSI-III) [29] for the assessment of verbal and performance cognitive abilities. Developmental history was also retrieved from medical records and caregivers’ interviews, including the history of developmental language delays, neuropsychiatric services received, the presence of an individualized education plan (IEP) or personalized didactic plan (PDP) and school attendance schedules (full-time or reduced). The presence of other certified diagnoses (e.g., specific learning disorders, cognitive disability, language delays, epilepsy, behavioral disorders), the use of psychopharmacological treatment and the presence of educational and psychotherapeutic support were also collected.

### 2.4. Statistical Analysis

Descriptive statistics were applied to all study variables. Given the small sample size and the non-normal distribution of quantitative variables, as confirmed by the Shapiro–Wilk test, medians and interquartile ranges (IQRs) are reported; frequencies and percentages were used for categorical data. For analytical purposes, questionnaire scores were categorized into three qualitative ranges (normative, borderline and clinical), and cognitive indices were dichotomized as follow: FSIQ < 85 vs. FSIQ ≥ 85 (i.e., −1 SD from the normative mean). To conduct exploratory, nominal association analyses, children were classified into four groups based on the predicted effects of *DMD* gene mutations on brain dystrophin isoform expression [30].

(a) Group 1 (Dp427−/Dp140+/Dp71+): mutations located upstream of intron 44;
(b) Group 2 (Dp427−/Dp140−/Dp71+): mutations located between exons 51 and 62;
(c) Group 3 (Dp427−/Dp140−/Dp71−): mutations involving exon 63 or downstream regions;
(d) Group 4 (Dp427−/Dp140 undetermined/Dp71+): mutations located between exons 45 and 51—a region in which the effect on Dp140 expression cannot be reliably predicted, as the Dp140 promoter is in intron 44, while its translation start site falls within exon 51. Children in this category were excluded from association analyses.

All analyses conducted were hypothesis-generating and exploratory in nature. The analyses examined relationships between the mutation group and neurocognitive, behavioral and clinical variables, as well as associations between ED indices, symptom severity, age and history of language delays. Ambulatory status was not entered into inferential testing but was compared descriptively between subgroups. Fisher’s exact test was applied for comparisons of qualitative data due to the small sample size. Considering the small sample size and the exploratory purpose, no correction for multiple comparisons was applied, and no regression analyses were performed; *p*-values are nominal and exploratory. Firth bias-reduced penalized logistic regression was used to estimate the odds ratio and its 95% profile penalized-likelihood confidence interval in association with the *p*-values. Statistical significance was set at *p* < 0.05 (two-tailed). Analyses were performed using IBM SPSS Statistics, version 26 (IBM Corp., Armonk, NY, USA) and R (version 4.5.2, 31 October 2025).

## 3. Results

### 3.1. Clinical, Genetic and Psychosocial Characteristics

The cohort consisted of 21 children with DMD, with a median age of 11 years [IQR 6]. The median age at diagnosis was 4 years [IQR 2]. Within the sample, 10 children presented an out-of-frame deletion (47.6%), five (23.8%) a point mutation and six (28.6%) a duplication in the *DMD* gene. According to the predicted effects on brain dystrophin isoform expression, most children belonged to Group 1 (Dp427−/Dp140+/Dp71+), representing 66.7% (14/21), while Group 2 (Dp427−/Dp140−/Dp71+) included five cases (23.8%). No child was classified as Group 3 (Dp427−/Dp140−/Dp71−). Finally, two variants (9.5%) were not classifiable, as they were located between exons 45 and 51—a region where the effect on Dp140 expression is difficult to predict accurately. The median age at steroid initiation was 5 years [IQR 5.7]. At enrollment, all children were receiving deflazacort as a standard corticosteroid therapy. Notably, six children (28.6%) in the cohort were non-ambulatory (i.e., a child unable to walk greater than 10 m independently).

At the time of enrollment, only five children (23.8%) had at least one documented neuropsychiatric comorbidity; among these, one child presented multiple diagnoses, while each of the remaining four children had exactly one comorbidity. In particular, the patient presenting multiple diagnoses had a specific learning disorder, intellectual disability and mixed conduct and emotional disorder. The remaining four patients showed intellectual disability (n = 1, 4.8%), a specific learning disorder (n = 1, 4.8%) and a language disorder (n = 2, 9.5%). In the cohort, the data showed that 11 children (52.4%) had experienced a delay in language acquisition and development. Regarding school interventions, 15 children (71.4%) attended school full-time, while six (28.6%) followed a reduced schedule. Most children (n = 17, 81.0%) benefited from the support of a special education teacher, and 16 out of 20 (80.0%) children had an individualized education plan (IEP). Regarding therapeutic interventions, three children (14.3%) received educator support and nine (42.9%) undertook a course of psychological support, while only two children (9.5%) were prescribed psychopharmacological treatment. Full sample demographics and clinical characteristics are summarized in Table 1.

### 3.2. Cognitive Functioning

Cognitive functioning data were retrospectively collected and were available for 11 of 21 children (52.4%). The median age at assessment was 8 years [IQR 3]. Cognitive functioning was assessed using age-appropriate Wechsler scales: WISC-IV in 8/11 (72.7%) and WPPSI-III in 3/11 (27.3%). To ensure comparability across participants, only the Full-Scale Intelligence Quotient (FSIQ) was considered. Among these 11 children, the FSIQ was ≥85 in 6/11 (54.5%), while it was <85 in five (45.5%); among these, 3/11 (27.3%) presented an FSIQ between 70 and 84, and 2/11 (18.2%) presented values below 70.

### 3.3. Psychometric Screening Test Scores

#### 3.3.1. Autism Spectrum Disorder-Related Features

ASD-related features were assessed using both parent-report measures (SCQ) and a clinician-administered structured interview (CASD).

On the SCQ (n = 20), one child (5.0%) reached the clinical cutoff (score ≥ 15); this child carried a deletion of exons 46–50 and was excluded from the ASD features versus cognitive functioning analyses due to the uncertainty in predicting its effect on Dp140 expression.

On the CASD (n = 20), scores were above the normative threshold in 6 out of 20 children (30.0%); specifically, four (20.0%) fell within the at-risk range, one (5.0%) in the borderline range and one (5.0%) in the clinical range (score = 16).

At the individual level, the participant who screened positive for ASD-related features on the SCQ scored within the normative range on the CASD. Conversely, all six participants who scored outside the normative range on the CASD presented negative SCQ screening results. These findings are reported descriptively, and no formal agreement analysis was performed.

Overall, 5.0% of participants scored above the clinical cutoff on the SCQ, whereas 30.0% scored outside the normative range on the CASD. However, in the absence of a matched control group and diagnostic confirmation, the results are presented descriptively and only indicate the presence of ASD-related screening-positive features within the study cohort.

#### 3.3.2. Attention-Deficit/Hyperactivity-Related Features

ADHD-related features were assessed using the following screening tools: parent report (ADHD-RS-V—parent version, CPRS-R and CBCL) and clinician-administered structured interviews (K-SADS ADHD module).

On the ADHD-RS-V—parent version (n = 19), one child (5.3%) exceeded the clinical cutoff on the inattention subscale, while no child reached the cutoff on the hyperactivity subscale.

On the CPRS-R (n = 20; scores > 61 considered out of normal range), inattentive features were more prominent than hyperactive–impulsive ones for ADHD-specific subscales: three children (15.0%) scored in the clinical range and two (10.0%) in the borderline range on the DSM-IV inattention subscale; one child (5.0%) scored in the clinical range and four (20.0%) in the borderline range on the ADHD index. Only three children (15.0%) scored within borderline range on the DSM-IV hyperactivity subscale, with no child reaching the clinical threshold.

Notably, both DSM-IV symptom subscales for inattention and hyperactivity (based on a symptom count threshold of ≥6 out of 9 criteria) were within the normative range for all children, indicating that no child met the full symptom burden required for a DSM-IV ADHD diagnosis on either dimension.

On the CBCL syndrome scales (n = 20; scores > or = 65 considered out of normal range), on the attention problems subscale, only two children (10.0%) scored in the borderline range and none in the clinical range, while, on the CBCL DSM-oriented ADHD problems subscale, all children’s scores were within the normative range.

On the K-SADS ADHD module (n = 20), one child (5.0%) exceeded the clinical cutoff on the inattention subscale, while none reached the threshold for the hyperactivity subscale.

Overall, ADHD-related screening findings were more prominent for inattention than for hyperactivity. Clinical-range inattentive scores were observed in 5–15% of participants across measures, while borderline inattentive scores ranged from 10% to 20%. No child scored in the clinical range for hyperactivity, whereas 15% showed borderline hyperactive features on the CPRS-R.

At the individual level, the participant who screened positive for inattention on the K-SADS also had clinical-range scores on the CPRS-R DSM-IV inattention scale, DSM-IV total scale and ADHD index, although the ADHD-RS-V score remained below the clinical threshold. Two additional participants presented clinical-range scores on the CPRS-R DSM-IV inattention scale, despite negative K-SADS findings; one of these participants also screened positive for inattention on the ADHD-RS-V. These findings are reported descriptively, and no formal agreement analysis was performed.

#### 3.3.3. Emotional–Behavioral Functioning

Emotional–behavioral problems were assessed through two complementary parent-report instruments, the CBCL and the CPRS-R, which provide a broad screening of psycho-emotional, social and behavioral difficulties at developmental age.

On the CBCL (n = 20), 16 children (80.0%) scored in the clinical or borderline range on the total competence scale. Internalizing difficulties were the most prominent finding, with withdrawal/depression affecting seven children (35.0%), somatic problems affecting five (25.0%) and anxiety/depression affecting four (20.0%). The 2007 scales identified post-traumatic stress problems in four children (20.0%) and obsessive–compulsive problems in two (10%).

On the CPRS-R (n = 20), the most affected domains were psycho-emotional and social: social problems affected nine children (45.0%), perfectionism affected seven (35.0%), anxiety/shyness affected six (30.0%) and psychosomatic problems affected five (25.0%).

Overall, the results from both questionnaires identified internalizing problems as the predominant area of difficulty. Across both instruments, a significant proportion of children showed clinical or borderline scores on scales related to perfectionism and obsessive–compulsive symptoms (seven children, 35.0% and two children, 10.0%, respectively).

#### 3.3.4. Executive Function Profile

Executive function was assessed through the BRIEF-2 Parent Version (n = 17). The Behavioral Regulation Index (BRI) scores were predominantly within the normative range, with only one child (5.9%) with scores in the clinical range. Subscales contributing to the BRI showed a similar pattern: inhibition and self-monitoring scores were within the normal range in, respectively, 14 (82.4%) and 15 children (88.2%), with no more than two children per subscale scoring above the normative threshold.

#### 3.3.5. Emotional Dysregulation Profile

Emotional dysregulation was assessed through two complementary approaches: the Emotional Regulation Index (ERI) of the BRIEF-2 and the CBCL Dysregulation Profile (DP). On the BRIEF-2 ERI (n = 17), six children (35.3%) scored above the normative threshold, with one child (5.9%) in the clinically elevated range. On the CBCL DP (n = 20), 3 children (15.0%) scored above the threshold for clinically relevant emotional dysregulation.

### 3.4. Exploratory Association Analyses

Considering cognitive functioning, only hypothesis-generating exploratory analyses were performed because of the small sample size (11/21), which was further reduced by the exclusion of 2 out of 11 patients who were classified into predicted dystrophin mutation Group 4 (Dp427−/Dp140 undetermined/Dp71+). The final analyses were thus conducted in only 9/21 (42.8%) patients. A nominal association emerged between the FSIQ and the predicted dystrophin mutation group, with a higher proportion of patients with FSIQ < 85 in the Dp140−/Dp71+ subgroup (4/4, 100.0%) compared to the Dp140+/Dp71+ subgroup, in which only one scored below 85 (1/5, 20.0%, nominal unadjusted *p* = 0.048, OR 27.0, profile penalized-likelihood 95% CI 1.50–4548.83, Fisher’s exact test). While multivariable regression was not feasible, potential confounders were descriptively analyzed between Group 1 and Group 2. The results of this analysis are summarized in Table 2.

Moreover, in the cohort, a higher proportion of patients with a positive history of developmental language delay emerged in the FSIQ < 85 subgroup (4/5, 80.0%) compared to the FSIQ ≥ 85 subgroup, in which only 1/6 (16.7%) presented a positive history.

Considering ASD-related features, CASD results were investigated in 18 children with available data. Children with CASD scores falling within the borderline, at-risk or clinical range were considered positive for ASD-related features. Again, only hypothesis-generating exploratory analyses were performed. In mutation Group 1 (Dp140+/Dp71+), a higher proportion of children (11 out of 13, 84.6%) reported scores within the normative range compared to mutation Group 2 (Dp140−/Dp71+), in which 60.0% (3 out of 5) presented scores in the at-risk/borderline/clinical range (nominal unadjusted *p* = 0.099, OR 6.44, profile penalized-likelihood 95% CI 0.82–64.9, Fisher’s exact test). These findings are only descriptive and need to be confirmed in further longitudinal studies with larger cohorts. No association was found between the mutation group and ADHD-related features on either the CPRS-R or CBCL subscales.

## 4. Discussion

Recent evidence has reported neurocognitive impairments and an increased prevalence of neuropsychiatric comorbidities, including ASD, ADHD, OCD and anxiety–depressive symptoms, in children with DMD compared to the pediatric general population. This observational study involved the comprehensive screening of cognitive, neuropsychological and emotional–behavioral difficulties of a monocentric cohort of children with DMD.

Regarding cognitive functioning, the data of 11/21 (52.4%) children were retrospectively collected in the cohort, and 9/21 (42.8%) were considered for analyses. In this subgroup, higher FSIQ scores were found in the Dp140+/Dp71+ brain dystrophin isoform status group compared with the Dp140−/Dp71+ group in the nominal association analysis conducted. This finding is limited by the very small subgroup of patients with available FSIQ data and thus by the possibility of selection bias; however, if confirmed in a larger cohort, it is consistent with recent evidence that indicates a potential protective role of Dp140 [11,31].

Considering ASD-related features, among the 20/21 (95.2%) children with available SCQ and CASD data, one child (5.0%) exceeded the normative cutoff on the SCQ, while six children (30.0%) scored above the normative threshold on the CASD. These results suggest the need for further diagnostic assessment to confirm the presence of an ASD diagnosis in participants who screened positive, since recent evidence highlights an increased prevalence of ASD-related features in children with DMD compared with the general pediatric population [8,32]. Descriptively, a higher proportion of ASD-related features on the CASD was found in the Dp140−/Dp71+ brain dystrophin isoform mutation group; this finding is only hypothesis-generating and needs to be confirmed and further investigated in larger cohorts.

Regarding ADHD-related features, although no participant met the DSM symptom count threshold for ADHD, attentional difficulties were predominant and consistently identified across all four tests administered. No child had clinical-range scores on hyperactivity subscales across measures. These results suggest the need to further investigate patients who exceed the normative ranges to confirm the presence of an ADHD diagnosis, since the literature highlights an increased vulnerability to inattention among children with DMD, with ADHD occurring more frequently than observed in the general pediatric population [11,31,32,33,34,35]. Notably, attentional difficulties do not appear to be secondary to disease progression or its psychological impact, as they have been documented from the earliest stages of the disease, supporting a primary neurobiological role of dystrophin deficiency in attentional circuits [36,37]. Conversely, the absence of clinically significant hyperactivity scores may reflect the unique clinical features of DMD. Progressive muscle degeneration and early fatigability could limit the expression of hyperactive behaviors, thereby reducing the likelihood of detecting symptoms typically characterized by excessive motor activity.

Emotional–behavioral screening tools highlighted the presence of obsessive–compulsive features; again, further diagnostic confirmation could be important in suspected cases, in light of previous reports of OCD-related symptomatology in DMD [32,38,39]. Although poorly investigated, the available studies report a higher prevalence of OCD symptoms in DMD (around 4.7%) [32,38] compared with the general population, where the prevalence is estimated at around 1–3%, although not always reaching a definitive diagnosis. In the present study, no specific instruments for OCD were used; however, both the CBCL and CPRS-R questionnaires revealed a significant proportion of children with clinical or borderline scores on the subscales related to perfectionism and obsessive–compulsive symptoms (35.0% and 10.0%, respectively). Although these findings are insufficient to support a formal diagnosis, they underscore the importance of systematically assessing this domain during periodic evaluations.

Beyond OCD, 16 out of 20 children (80.0%) obtained clinical or borderline scores on at least one scale related to internalizing problems. Specifically, the CPRS-R and CBCL questionnaires identified scores above the normative threshold in the dimensions of anxiety/shyness/depression (overall frequency between 20 and 30%), social withdrawal (35.0%), somatic problems (15–25%) and difficulties in social adjustment (45.0%). In the literature, an increased risk of internalizing disorders in children with DMD is largely documented [10]. The estimated prevalence for anxiety and depression is 27% [34,38], with higher values in non-ambulatory individuals. Although the results observed in the cohort do not constitute a clinical diagnosis, they highlight the importance of ongoing monitoring and targeted assessments to identify internalizing disorders early in children with DMD, in order to enable timely complete diagnosis and personalized intervention in at-risk cases [17].

Moreover, emotional regulation difficulties emerged as a transversal area of possible vulnerability in the cohort. G. Masi et al. [40] proposed that emotional dysregulation may represent a neurodevelopmental disorder or, alternatively, a shared transdiagnostic risk factor underlying the co-occurrence of internalizing and externalizing problems. This framework appears particularly relevant in DMD, where emotional and behavioral dysregulation has been reported as the most frequent neurodevelopmental symptom, affecting up to 38.7% of children in a large cohort of 700 boys [41].

Upon retrospectively analyzing other possible comorbidities in the cohort, the presence of certified neuropsychiatric diagnoses confirmed the variability and complexity of the neuropsychological profile of children with DMD. Approximately one quarter of children (23.8%) presented at least one comorbidity, including cases of specific learning disorders, cognitive disability, language disorders and mixed conduct and emotional disorder. Particularly noteworthy is the proportion of children with a history of developmental language delays, found in more than half of cases (52.4%), consistent with previous reports of frequent impairments in language skills [42], which may manifest in the early years of life with delays in the acquisition of first words and phrases, followed by learning difficulties, particularly in reading, writing and arithmetic tasks [39].

Additionally, the analysis of school and therapeutic interventions received by the children in this cohort suggests good adherence to the available educational support system: most children attended school full-time and benefited from a support teacher and an individualized educational plan (IEP), with particular attention to children with FSIQ < 85, for whom support measures appear adequate. This confirms the importance of school inclusion and personalized educational support as fundamental tools to foster adaptation and learning in children with DMD [6,7]. The scenario appears different, however, when considering educational and emotional–behavioral interventions: specifically, only three children (14.3%) received an educational intervention, while less than half had access to psychological support. The use of psychopharmacological treatment was limited to two cases (9.5%). Notably, only a minority of children with emotional dysregulation or clinically relevant internalizing symptomatology were receiving targeted interventions, suggesting a discrepancy between the needs identified through the screening psychometric assessments and the services provided.

According to the standard of care (SoC) for DMD [14], an informal screening of mental health and quality of life should be performed at each routine visit. Tools suggested for the assessment are the Strengths and Difficulties Questionnaire (SDQ) for children with DMD and the Personal Adjustment and Role Skill Scale III Edition (PARSIII) for caregivers. The multimodal battery proposed in this study, although not formally validated, was shown to be feasible; it addresses the SoC indication and may broaden screening for major neuropsychiatric disorders in children with DMD, particularly considering recent evidence of neuropsychological involvement. The SoC indicates that neuropsychological tests should be performed when there is a risk or clinical suspicion of neuropsychiatric disorders, with re-assessment every 2–3 years. In light of recent evidence, and considering the specific characteristics of the developmental age, we suggest and propose to screen for neuropsychiatric disorders at key disease-stage transitions (e.g., one year from diagnosis and at school grade transitions for cognitive assessment; at school grade transitions, at loss of ambulation and before the transition to adult services for neuropsychosocial assessment). The child’s neuropsychiatrist or neuropsychologist should be considered whenever screening results or parental/teacher concerns suggest clinically significant difficulties in order to plan a confirmatory diagnosis, treatment and follow-up.

## 5. Limitations

This study presents several methodological limitations.

First, parent-report screening instruments, although validated, do not allow for definitive clinical diagnoses and may introduce caregiver-related reporting bias, with risks of both underestimation and overestimation of symptoms. The absence of self-report measures, directly completed by children, further limits the perspectives captured, particularly for older adolescents capable of providing reliable self-assessments.

Second, no standardized clinical assessment by a child and adolescent neuropsychiatrist was systematically performed, which would be necessary to confirm the screening findings and formulate formal diagnoses.

Third, teacher-report instruments were not included, precluding the assessment of difficulties in the school setting—a relevant context for the detection of both attentional and behavioral problems.

Fourth, the cross-sectional design does not allow for the observation of symptom trajectories over time, nor for the disentanglement of neurodevelopmental disease progression-related contributions to the observed psychopathological profile.

Fifth, FSIQ data were available for only 11 out of 21 children, limiting the representativeness of the cognitive findings. The descriptive comparison between the subgroup with available FSIQ data versus the subgroup without FSIQ available data showed differences, particularly in the dystrophin isoform expression pattern distribution, school and educational support and psychopharmacological treatment. These characteristics were more frequently observed in the subgroup with available FSIQ data. This pattern may suggest that children with greater clinical or educational concerns were more likely to undergo cognitive assessment. Conversely, the absence of school support among participants without available FSIQ data may indicate a lower level of previously recognized cognitive difficulties, although this cannot be assumed with certainty. Notably, 9 of the 10 participants without available FSIQ data belonged to brain dystrophin isoform status Group 1, which was associated in our cohort with a lower burden of cognitive difficulties. This finding is consistent with, but does not prove, the hypothesis that cognitive testing may be less frequently requested in children without evident clinical concerns. Still, this apparent consistency cannot be considered confirmatory and, therefore, missing cognitive data cannot be considered random, and the possibility of selection bias remains (Supplementary Material Table S1).

Sixth, although standardized scores were used to allow comparisons across age groups, the use of different age-appropriate cognitive instruments may have introduced a degree of measurement bias, as psychometric properties are not always fully comparable across versions designed for different developmental stages; this may have led to the relative over- or underestimation of cognitive performance in some age subgroups.

Finally, the small sample size—further reduced by the absence of Group 3 children and the exclusion of two undetermined mutations—significantly limited the statistical power and the generalizability of the findings, namely regarding the results of the exploratory association analyses. Potential confounders such as age, ambulatory status, disease severity, school support and previous neuropsychiatric diagnoses were not formally assessed, as the small sample size precluded adequately powered multivariable analyses; their possible influence on the cognitive and emotional–behavioral outcomes cannot therefore be excluded and should be addressed in future, adequately powered studies. However, potential confounders for which data were available were analyzed descriptively to improve the interpretability of the results. Disease severity was assessed only by ambulatory status; motor functional scales were not collected.

## 6. Conclusions

This study contributes to the still-limited evidence regarding the neuropsychological and emotional–behavioral burden experienced by children with DMD beyond motor impairment. These difficulties are frequently underrecognized and undertreated, despite their substantial impacts on the quality of life of both children and caregivers. Overall, attentional difficulties, internalizing problems and emotional dysregulation features emerged at screening in the cohort, highlighting the relevance of the psychopathological dimension in DMD, even in the absence of a formal neuropsychiatric diagnosis. Moreover, cognitive functioning was heterogeneous across the cohort, ranging from normal FSIQ scores to intellectual disability. However, the lack of a matched control group, together with the cross-sectional, observational nature of the design, the small sample size and limited data availability, restricts the study results. If confirmed in longitudinal studies with larger cohorts, these findings may support the value of implementing systematic, standardized cognitive, neuropsychological and emotional–behavioral screening for children with DMD from the time of diagnosis, aimed at facilitating personalized multidisciplinary care pathways and optimizing psychoeducational and psychological interventions to reduce the disease burden.

## Figures and Tables

**Table 1:** Demographic, clinical and neuropsychiatric characteristics.

Variable	Overall Sample (n = 21)
**Demographics**	
Age, years, median [IQR]	11.0 [6]
**Steroid therapy**	
Treatment initiation age, years, median [IQR]	4.9 [5.7]
Brain dystrophin isoform status *, n (%)	
Group 1 (Dp427−/Dp140+/Dp71+), n (%)	14 (66.7%)
Group 2 (Dp427−/Dp140−/Dp71+), n (%)	5 (23.8%)
Group 3 (Dp427−/Dp140−/Dp71−), n (%)	0 (0.0%)
Group 4 (Dp427−/Dp140 undetermined/Dp71+), n (%)	2 (9.5%)
**Neuropsychiatric comorbid diagnoses**	
≥1 comorbidity, n (%)	5 (23.8%)
Specific learning disorder, n (%)	1 (4.8%)
Intellectual disability, n (%)	1 (4.8%)
Language disorder, n (%)	2 (9.5%)
Specific learning disorder, intellectual disability, mixed conduct and emotional disorder, n (%)	1 (4.8%)
**Developmental history**	
Language delay, n (%)	11 (52.4%)
**School interventions**	
Reduced schedule, n (%)	6 (28.6%)
Special education teacher, n (%)	17 (81.0%)
Individualized education plan (IEP), n (%) (* n = 20)	16 * (80.0%)
**Therapeutic interventions**	
Educator support, n (%)	3 (14.3%)
Psychological support, n (%)	9 (42.9%)
**Ambulatory n, %**	15 (71.4%)
**Psychopharmacological treatment**, n (%)	2 (9.5%)

Dp: dystrophin protein. * Classification based on Stimpson et al., 2022 [30].

**Table 2:** Potential confounders of association between FSIQ < 85 vs. FSIQ ≥ 85 and brain dystrophin isoform status—Group 1 vs. Group 2 *.

Variable	Group 1 (n = 14)	Group 2 (n = 5)
Age, years, median [IQR]	12 [6]	10 [4]
Neuropsychiatric comorbid diagnoses		
Specific learning disorder, n (%)	0 (0.0%)	1 (20.0%)
Language disorder, n (%)	1 (7.1%)	1 (20.0%)
Conduct disorder, n (%)	0 (0%)	0 (0.0%)
Special education teacher, n (%)	11 (78.6%)	4 (80.0%)
Educator support, n (%)	3 (21.4%)	0 (0.0%)
Ambulant n, %	10 (71.4%)	4 (80.0%)

* Classification based on Stimpson et al., 2022 [30].

## Data Availability

The original contributions presented in this study are included in the article/Supplementary Materials. Further inquiries can be directed to the corresponding author.

## Supplementary Materials

The following supporting information can be downloaded at https://www.mdpi.com/article/10.3390/children13080992/s1. Table S1: Descriptive comparison between IQ available data subgroup vs. IQ not available data.

## References

[1] Duan D., Goemans N., Takeda S., Mercuri E., Aartsma-Rus A. (2021). Duchenne muscular dystrophy. Nat. Rev. Dis. Prim..

[2] Crisafulli S., Sultana J., Fontana A., Salvo F., Messina S., Trifirò G. (2020). Global epidemiology of Duchenne muscular dystrophy: An updated systematic review and meta-analysis. Orphanet J. Rare Dis..

[3] Bushby K., Finkel R., Birnkrant D.J., Case L.E., Clemens P.R., Cripe L., Kaul A., Kinnett K., McDonald C., Pandya S. (2010). Diagnosis and management of Duchenne muscular dystrophy, part 1: Diagnosis, and pharmacological and psychosocial management. Lancet Neurol..

[4] Mendell J.R., Ms C.S., Leslie N.D., Flanigan K., Al-Dahhak R., Gastier-Foster J., Kneile K., Dunn D.M., Duval B., Aoyagi A. (2012). Evidence-based path to newborn screening for duchenne muscular dystrophy. Ann. Neurol..

[5] Birnkrant D.J., Bushby K., Bann C.M., Alman B.A., Apkon S.D., Blackwell A., Case L.E., Cripe L., Hadjiyannakis S., Olson A.K. (2018). Diagnosis and management of Duchenne muscular dystrophy, part 2: Respiratory, cardiac, bone health, and orthopaedic management. Lancet Neurol..

[6] D’Alessandro R., Ragusa N., Vacchetti M., Rolle E., Rossi F., Brusa C., Davico C., Vitiello B., Mongini T., Ricci F.S. (2021). Assessing Cognitive Function in Neuromuscular Diseases: A Pilot Study in a Sample of Children and Adolescents. J. Clin. Med..

[7] Biagi L., Lenzi S., Cipriano E., Fiori S., Bosco P., Cristofani P., Astrea G., Pini A., Cioni G., Mercuri E. (2021). Neural substrates of neuropsychological profiles in dystrophynopathies: A pilot study of diffusion tractography imaging. PLoS ONE.

[8] Keselman D., Glanzman A., Thelen M.Y., Prosser L.A., McGuire J., Matesanz S.E. (2025). Motor function testing rates and outcomes in Duchenne muscular dystrophy with comorbid autism and attention-deficit/hyperactivity disorder. Neuromuscul. Disord..

[9] Doorenweerd N., Mahfouz A., van Putten M., Kaliyaperumal R., Hoen P.A.C.T., Hendriksen J.G.M., Aartsma-Rus A.M., Verschuuren J.J.G.M., Niks E.H., Reinders M.J.T. (2017). Timing and localization of human dystrophin isoform expression provide insights into the cognitive phenotype of Duchenne muscular dystrophy. Sci. Rep..

[10] Weerkamp P.M.M., Geuens S., Collin P., Goemans N., Vermeulen R.J., De Waele L., Hendriksen J.G., Klinkenberg S. (2023). Psychopharmaceutical treatment for neurobehavioral problems in Duchenne muscular dystrophy: A descriptive study using real-world data. Neuromuscul. Disord..

[11] Snow W.M., Anderson J.E., Jakobson L.S. (2013). Neuropsychological and neurobehavioral functioning in Duchenne muscular dystrophy: A review. Neurosci. Biobehav. Rev..

[12] Hoskens J., Paulussen S., Goemans N., Feys H., De Waele L., Klingels K. (2024). Early motor, cognitive, language, behavioural and social emotional development in infants and young boys with Duchenne Muscular Dystrophy—A systematic review. Eur. J. Paediatr. Neurol..

[13] Chesshyre M., Ridout D., Hashimoto Y., Ookubo Y., Torelli S., Maresh K., Ricotti V., Abbott L., Gupta V.A., Main M. (2022). Investigating the role of dystrophin isoform deficiency in motor function in Duchenne muscular dystrophy. J. Cachexia Sarcopenia Muscle.

[14] Birnkrant D.J., Bushby K., Bann C.M., Apkon S.D., Blackwell A., Colvin M.K., Cripe L., Herron A.R., Kennedy A., Kinnett K. (2018). Diagnosis and management of Duchenne muscular dystrophy, part 3: Primary care, emergency management, psychosocial care, and transitions of care across the lifespan. Lancet Neurol..

[15] Hoskin J., James B., Finch J. (2025). ‘*More than a just a physical condition*’—Recognising the educational and emotional needs of children and young adults with duchenne muscular dystrophy. Eur. J. Spec. Needs Educ..

[16] Bever A., Audhya I., Szabo S.M., Mickle A., Feeny D., Malone D., Neumann P., Iannaccone S., Gooch K. (2024). ‘You Take This Day by Day, Come What May’: A Qualitative Study of the Psychosocial Impacts of Living with Duchenne Muscular Dystrophy. Adv. Ther..

[17] Gadaleta G., Geagan C., Schiava M., Riguzzi P., Hendriksen J., Campbell C., McDermott M.P., Martens W.B., Gregory S.J., Mongini T.E. (2026). Neurobehavioral Profiles in Young Steroid-Naive Boys With Duchenne Muscular Dystrophy: A Baseline Data Analysis From the FOR-DMD Trial. Neurology.

[18] Miranda R., Weerkamp P.M.M., Kolesnik A., Geagan C., Chieffo D., Suárez-Bagnasco M., Skuse D., Vroom E., Niks E.H., Vissing J. (2026). Screening for brain-related comorbidities in Duchenne muscular dystrophy: Construction, reliability, and validity of the BIND screener. Dev. Med. Child Neuro.

[19] Truba N., Sorensen S., Bearden R., Haley B., Spray B., Kinnett K., Schrader R., Veerapandiyan A., Colvin M.K. (2025). The BELS questionnaire: A novel screening tool for neurodevelopmental and psychiatric symptoms in pediatric dystrophinopathy. Muscle Nerve.

[20] Achenbach T.M., Rescorla L.A. (2001). Manual for the ASEBA School-Age Forms & Profiles.

[21] Conners C.K. (2007). Conners’ Rating Scales—Revised: Manuale.

[22] DuPaul G.J., Power T.J., Anastopoulos A.D., Reid R.C. (2016). ADHD Rating Scale—5 for Children and Adolescents: Checklists, Norms, and Clinical Interpretation.

[23] Allen C.W., Silove N., Williams K., Hutchins P. (2007). Validity of the social communication questionnaire in assessing risk of autism in preschool children with developmental problems. J. Autism Dev. Disord..

[24] Gioia G.A., Guy S.C., Kenworthy L. (2017). Behavior Rating Inventory of Executive Function. J. Pediatr. Neuropsychol..

[25] Marano A., Innocenzi M., Devescovi A., D’Amico S. (2016). BRIEF 2: Behavior Rating Inventory of Executive Function. Adattamento Italiano.

[26] Mayes S.D. (2012). Checklist for Autism Spectrum Disorder (CASD).

[27] Sogos C., Di Noia S.P., Fioriello F. (2019). K-SADS-PL DSM-5. Intervista Diagnostica per la Valutazione dei Disturbi Psicopatologici in Bambini e Adolescenti.

[28] Orsini A., Pezzuti L., Picone L. (2012). WISC-IV. Contributo alla Taratura Italiana.

[29] Sannio Fancello G., Cianchetti C. (2008). WPPSI-III: Contributo alla Taratura Italiana.

[30] Stimpson G., Raquq S., Chesshyre M., Fewtrell M., Ridout D., Sarkozy A., Manzur A., Gupta V.A., De Amicis R., Muntoni F. (2022). Growth pattern trajectories in boys with Duchenne muscular dystrophy. Orphanet J. Rare Dis..

[31] Gillenstrand J., Broberg M., Kroksmark A.-K., Tulinius M., Ekström A.-B. (2025). Concordance between parental and self-rated and performance-based measures of executive functioning in boys with Duchenne muscular dystrophy: The role of parental life satisfaction and carrier versus non-carrier status. Child Neuropsychol..

[32] Hendriksen J.G.M., Vles J.S.H. (2008). Neuropsychiatric Disorders in Males With Duchenne Muscular Dystrophy: Frequency Rate of Attention-Deficit Hyperactivity Disorder (ADHD), Autism Spectrum Disorder, and Obsessive—Compulsive Disorder. J. Child Neurol..

[33] Vaillend C., Aoki Y., Mercuri E., Hendriksen J., Tetorou K., Goyenvalle A., Muntoni F. (2025). Duchenne muscular dystrophy: Recent insights in brain related comorbidities. Nat. Commun..

[34] Pascual-Morena C., Cavero-Redondo I., Reina-Gutiérrez S., Saz-Lara A., López-Gil J.F., Martínez-Vizcaíno V. (2022). Prevalence of Neuropsychiatric Disorders in Duchenne and Becker Muscular Dystrophies: A Systematic Review and Meta-analysis. Arch. Phys. Med. Rehabil..

[35] Pane M., Lombardo M.E., Alfieri P., D’Amico A., Bianco F., Vasco G., Piccini G., Mallardi M., Romeo D.M., Ricotti V. (2012). Attention Deficit Hyperactivity Disorder and Cognitive Function in Duchenne Muscular Dystrophy: Phenotype-Genotype Correlation. J. Pediatr..

[36] De Moura M.C.D.S., Valle L.E.R.D., Resende M.B.D., Reed U.C., Pinto K.O. (2010). Visuospatial attention disturbance in Duchenne muscular dystrophy. Dev. Med. Child Neuro.

[37] Chen W.-Y., Wilson P.H., Wu S.K. (2012). Deficits in the covert orienting of attention in children with Developmental Coordination Disorder: Does severity of DCD count?. Res. Dev. Disabil..

[38] Banihani R., Smile S., Yoon G., Dupuis A., Mosleh M., Snider A., McAdam L. (2015). Cognitive and Neurobehavioral Profile in Boys With Duchenne Muscular Dystrophy. J. Child Neurol..

[39] Pezzoni L., Brusa R., Difonzo T., Magri F., Velardo D., Corti S., Comi G.P., Saetti M.C. (2024). Cognitive abnormalities in Becker muscular dystrophy: A mysterious link between dystrophin deficiency and executive functions. Neurol. Sci..

[40] Masi G., Sesso G., Pfanner C., Valente E., Molesti A., Placini F., Boldrini S., Loriaux N., Drago F., Montesanto A.R. (2021). An Exploratory Study of Emotional Dysregulation Dimensions in Youth With Attention Deficit Hyperactivity Disorder and/or Bipolar Spectrum Disorders. Front. Psychiatry.

[41] Darmahkasih A.J., Rybalsky I., Tian C., Shellenbarger K.C., Horn P.S., Lambert J.T., Wong B.L. (2020). Neurodevelopmental, behavioral, and emotional symptoms common in Duchenne muscular dystrophy. Muscle Nerve.

[42] Chieffo D.P.R., Moriconi F., Mastrilli L., Lino F., Brogna C., Coratti G., Altobelli M., Massaroni V., Norcia G., Ferraroli E. (2022). Language Development in Preschool Duchenne Muscular Dystrophy Boys. Brain Sci..

