# Supplementary material for: Neuropsychological and Emotional–Behavioral Profiles in Pediatric Duchenne Muscular Dystrophy: A Single-Center Clinical Study
Source: Children (Basel). 2026 Jul 27;13(8):992. doi: 10.3390/children13080992 (PMC13511711; doi:10.3390/children13080992)
Supplement: Supplementary file 1 [file children-13-00992-s001.zip › children-4403380-supplementary.pdf]

Table S1. Descriptive comparison between IQ available data subgroup vs. IQ not available data.

**Variables**	**IQ Available (n=11)**	**IQ Not Available (n=10)**
Age median, [IQR]	13 [5]	10 [5]
Ambulatory n, %	8 (72.7%)	7 (70.0%)
Dystrophin isoform group *		
- Group 1 n, %	5 (45.5%)	9 (90.0%)
- Group 2 n, %	4 (36.4%)	1 (10.0%)
- Group 3 n, %	0 (0.0%)	0 (0.0%)
- Group 4 n, %	2 (18.2%)	0 (0.0%)
History of Language delay n, %	5 (45.5%)	6 (60.0%)
Presence of School Support n, %	2 (18.2%)	0 (0.0%)
Presence of Educational Support n, %	4 (36.4%)	1 (10.0%)
Presence of Neuropsychiatric Diagnoses n, %		
- Specific Learning Disorder	2 (18.2%)	0 (0.0%)
- Conduct disorder	1 (9.1%)	1 (10.0%)
- Language disorder	1 (9.1%)	1 (10.0%)
Psychopharmacological treatment	2 (18.2%)	0 (0.0%)

* Classification based on Stimpson et al., 2022 [30]
